# Exploring the Control of PARP1 Levels in High-Grade Serous Ovarian Cancer

**DOI:** 10.3390/cancers15082361

**Published:** 2023-04-18

**Authors:** Giuseppina Raspaglio, Marianna Buttarelli, Natalia Cappoli, Alessandra Ciucci, Anna Fagotti, Giovanni Scambia, Daniela Gallo

**Affiliations:** 1 Unità di Medicina Traslazionale per la Salute della Donna e del Bambino, Dipartimento Scienze della Salute della Donna, del Bambino e di Sanità Pubblica, Fondazione Policlinico Universitario A. Gemelli, IRCCS, 00168 Roma, Italy; 2 Dipartimento Universitario Scienze della Vita e Sanità Pubblica—Sezione di Ginecologia ed Ostetricia—Università Cattolica del Sacro Cuore, 00168 Roma, Italy; 3 Dipartimento Scienze della Salute della Donna, del Bambino e di Sanità Pubblica, Fondazione Policlinico Universitario A. Gemelli, IRCCS, 00168 Roma, Italy

**Keywords:** personalized medicine, STAT1, interferon-ɣ

## Abstract

**Simple Summary:**

In high-grade serous ovarian cancer (HGSOC), the PARP (poly(ADP-ribose) polymerase) inhibitor resistance limits the therapeutic strategies. Understanding the regulation of PARP1 expression in HGSOC may be functional to overcome this issue. We recently demonstrated that, in cervical cancer cells, STAT1 (Signal Transducer and Activator of Transcription) controls PARP1 levels, also interacting with STAT3. Hence, we evaluated the possibility that the same mechanism may occur in HGSOC. The STAT1/STAT3 effects on PARP1 were studied in both established and primary HGSOC cells. Results suggest that STAT1 might act at both transcriptional and post-transcriptional levels to modulate PARP1 levels. Remarkably, bioinformatics analysis of databases revealed that higher levels of STAT1 correlate with positive outcomes, as well as with good responses to chemotherapy in HGSOC patients. These results indicate that new molecular interactions have to be studied in order to solve the PARP inhibitor resistance and to improve personalized therapeutic approaches in HGSOC.

**Abstract:**

High-grade serous ovarian cancer (HGSOC) is a leading cause of mortality from gynecologic malignancies worldwide. Although a transformative improvement has been shown with the introduction of PARP (poly(ADP-ribose) polymerase) inhibitors, the emergence of resistance to these drugs represents a therapeutic challenge. Hence, expanding our understanding of mechanisms behind the control of PARP1 expression can provide strategic guidance for the translation of novel therapeutic strategies. The Signal Transducer and Activator of Transcription (STAT) family of proteins consists of transcription factors critically involved in the regulation of important cellular functions. Notably, we recently demonstrated that, in cervical cancer cells, STAT1 controls PARP1 levels through multiple mechanisms, possibly involving also STAT3. Here, we tested the hypothesis that a similar mechanism might be operative in HGSOC. To this end, the impact of STAT1/STAT3 modulation on PARP1 expression was assessed in established and primary HGSOC cells, and molecular biology studies proved that STAT1 might act at both transcriptional and post-transcriptional levels to modulate the PARP1 level. Notably, bioinformatics analysis of TCGA databases demonstrated that increased STAT1 mRNA expression levels are associated with a favorable prognosis and with response to chemotherapy in HGSOC patients. Our findings suggest an alternative strategy for targeting HGSOC cells based on their dependency on PARP1.

## 1. Introduction

Ovarian cancer is a leading cause of morbidity and mortality from gynecologic malignancies worldwide with an estimated 207,252 deaths in 2020 worldwide [1]. The prognosis of epithelial ovarian cancer, accounting for 90% of ovarian cancer, worsens markedly by stage [2]. High-grade serous ovarian cancer (HGSOC) is the most prevalent histological type characterized by diagnosis at an advanced stage. Although the majority of patients initially respond to chemotherapy, they relapse and develop resistance within 2 years with less than one-half of women surviving beyond 5 years after diagnosis [3]. Progress in the development of targeted therapy including inhibitors of poly(ADP-ribose) polymerase (PARPi) has significantly improved outcome, mainly in patients harboring a BRCA (breast cancer gene) mutation [4,5]. The PARP gene superfamily consists of 17 members, with PARP1 and PARP2 being the most studied due to their involvement in DNA damage response (DDR) [6]. Homologous recombination deficiency (HRD), defined by either a BRCA1/2 mutation and/or genomic instability, is a major determinant of PARPi efficacy through synthetic lethality. Specifically, among PARPi, olaparib was initially approved for the maintenance treatment of recurrent platinum-sensitive BRCA1/2 mutant epithelial ovarian cancer, and then additional studies suggested higher benefits of PARPi maintenance, as monotherapy or in combination with bevacizumab, in mutated BRCA and HRD than HR-proficient (HRP) HGSOC patients [7]. However, although tumors in BRCA1/2 mutation carriers may initially respond to platinum and PARPi, many develop resistance over time, and this has proved to be a major problem in the clinic [8]. Despite having demonstrable benefits compared with chemotherapy alone, these drugs display several on- and off-target toxicities, which are shared by the pharmacological class or are drug specific [9]. In light of these considerations, expanding our understanding of the mechanisms behind the control of PARP1 expression can provide strategic guidance for the translation of novel therapeutic strategies, finally improving patient survival. 

The JAK-STAT (Janus kinases/signal transducers and activators of transcription) pathway has been recognized as one of the core cancer pathways in different cancers, including ovarian cancer [10,11]. Specifically, STAT3 was shown to be constitutively activated in ovarian cancer cells, and its continued activation is likely predictive of an unfavorable prognosis in patients [10,12]. On the other hand, with regard to STAT1, contradictory findings have been reported, suggesting a dual role with both antitumor and tumor-promoting activity [13]. Notably, clinical data support a protective role for STAT1 in HGSOC, with high intratumoral expression associated with an increased patient progression-free survival [14,15]. While STAT3 is predominantly activated by cytokines and growth factors [16], STAT1 is mainly activated by interferons but can also be activated to some extent by other cytokines, such as interleukins (e.g., IL-6) and growth factors [13].

We recently demonstrated that in cervical cancer cells (HPV-positive CaSki and C-4I), a perturbation in the balanced expression/activation of STAT1 and STAT3 might improve the efficacy of DNA-damaging treatments, at least partially, by lowering PARP1 levels in tumors [17]. Specifically, our data suggest that both transcriptional and post-transcriptional mechanisms might be involved in the role of STAT1/STAT3-mediated control of PARP1 levels [17]. With regard to post-transcriptional mechanisms, we demonstrated a crucial role of the proteasome subunits PA28α and PA28β, directly modulated by STAT1, accordingly to other findings [17,18]. In the present study, we test the hypothesis that a similar mechanism might be operative in HGSOC, with potential clinical implication in personalized cancer therapeutic approaches.

## 2. Materials and Methods

### 2.1. Established and Primary HGSOC Cells

Human ovarian cancer cell lines (COV318; NIH:OVCAR-3 and OV-90; HEY) were purchased from European Collection of Cell Cultures (ECACC, Salisbury, UK) and American Type Culture Collection (ATCC, Milan, Italy), and kindly donated from Susan Horwitz (Albert Einstein Medical College), respectively. The origin and characteristics of the established cell lines have been previously reported [19,20,21,22]. Briefly, COV318 is an ovarian serous cancer cell line isolated from peritoneal ascites [19]. The NIH:OVCAR-3 line was derived from the ascites of a patient treated with chemotherapy due to an ovarian adenocarcinoma [20]. OV-90 cells were isolated from a chemotherapy-naive grade 3, stage IIIC, malignant papillary serous adenocarcinoma [21]. The HEY cell line was obtained from human ovarian xenografts implanted originally from a patient with moderately differentiated papillary cystadenocarcinoma of the ovary [22]. NIH:OVCAR-3 and HEY cells were grown in RPMI 1640 (Sigma-Aldrich, Merck KGaA, Darmstadt, Germany), while COV318 was cultured in DMEM (Sigma Aldrich, St. Louis, MO, USA). OV-90 cells were maintained in 1:1 mixture of MCDB 105 medium (containing a final concentration of 1.5 g/L sodium bicarbonate) and Medium 199 (containing a final concentration of 2.2 g/L sodium bicarbonate). All media were supplemented with 10% FBS (15% for OV-90 cells) plus 1% MEM (Minimum Essential Medium) Non-Essential Amino Acid, 1 mM glutamine and 1% kanamycin. Cells were maintained in a fully humidified atmosphere of 5% CO_2_/95% air, at 37 °C. Cells were tested for mycoplasma (MycoAlert mycoplasma detection kit, LONZA, Rockland, ME, USA) and authenticated by STR (Short Tandem Repeat) DNA profiling.

Primary cancer cells were isolated from tissue samples of newly diagnosed and histologically confirmed HGSOC enrolled patients, admitted to the Gynaecologic Oncology Unit, Fondazione Policlinico Universitario A. Gemelli, IRCCS, Roma. Primary cancer cells were characterized by morphology, immunocytochemistry, and immunofluorescence as described previously [23]. The study was approved by the local Ethics Committee and Institutional Review Board (Protocol 19402/18 ID: 2045). All data were managed using anonymous numerical codes (OV.GEM). In detail, OV.GEM-9 was isolated from a 38-year-old woman diagnosed with HGSOC (FIGO stage IVB).

### 2.2. Reagents and Treatments

For silencing experiments, the following reagents were used: predesigned SMARTpool STAT1-targeting siRNA (siSTAT1), non-targeting control siRNA (siC) (Dharmacon, Lafayette, CO, USA) and TransFectin Lipid Reagent (Bio-Rad Laboratories, Hercules, CA, USA). Transfection was performed following suppliers’ guidelines.

IFN-γ and cisplatin (both from Sigma-Aldrich) were dissolved in distilled water. For single treatments, IFN-γ was used either at 10 ng/mL (OVCAR-3 and OV-90) or 20 ng/mL (OV.GEM). The selection of dosage and time of exposure was made based on previous data and on preliminary internal studies (data not shown) [17].

For combination studies, cells were preincubated with either IFN-γ (10 ng/mL) or complete culture medium for 24 h, and then treated with a 3 μM dose cisplatin (corresponding to IC50 in both cell lines after 72 h of treatment, as previously reported [23]).

### 2.3. Cytotoxicity Assay

OVCAR-3 and OV-90 cells were plated in a 96-well plate at a density of 6 × 10^3^/well, and the day after plating, cells were treated with IFN-γ and/or cisplatin as specified above. After treatment, Cell Counting Kit-8 (CCK8, Sigma Aldrich) was added into each well, according to the manufacturer’s instruction. After 1 h, the optical density (OD) of each experimental condition (in triplicate) was measured at 450 nm with a microplate reader (Enspire, Perkin Elmer, Walthman, MA, USA). The percentage of cell viability was determined using the OD mean of each experimental condition.

### 2.4. Chromatin Immunoprecipitation (ChIP)-qPCR Assay

ChIP-qPCR assay was performed to address the STAT1/STAT3 occupancy on the PARP1 promoter. OVCAR-3 and OV-90 cells at different conditions (treated with IFN-γ for 24/48 h or siSTAT1 for 48 h and respective controls) were fixed in 1% formaldehyde/PBS and, after 10 min, glycine (125 mM) was added. Briefly, cells were incubated in hypotonic buffer to isolate the nuclei. Then, the extracted genomic DNA was sonicated on ice, and precleared chromatin was subjected to STAT1/STAT3 specific antibody incubation overnight. After isolation of the immunocomplexes, qPCR assay was performed to amplify genomic regions in proximity to the putative STAT1/STAT3 binding sites. Two independent chromatin isolations were carried out, and two qPCR amplification replicates were used for quantitative analysis. Further details were previously described [17].

### 2.5. Luciferase Assay

To evaluate the luciferase activity of the PARP1 (NM_001618) promoter, previously described plasmids were used [17]. Briefly, OVCAR-3 and OV-90 cells were transfected with pGL3-Basic or pGL3-PARP1-P plus pRL-TK, taken as internal control, and after 24 h, cells were treated with IFN-γ (10 ng/mL) or distilled water. After an additional 24 h, the Dual Luciferase Reporter assay system (Promega, Madison, WI, USA) was used to measure luciferase activity.

### 2.6. Isolation of RNA

Total RNA was isolated from cells using the RNeasy RNA isolation kit (Qiagen, Milan, Italy), according to the manufacturer’s protocol and stored at −80 °C until analysis. The recovered RNA concentration was measured using the Nanodrop (Thermo Fisher Scientific, Waltham, MA, USA).

### 2.7. Real-time Quantitative PCR

Real-time qPCR on mRNAs was performed as previously described [17] using the primers listed in Table S1. The relative quantification of target mRNA was performed according to the ΔΔCt method [24].

### 2.8. Western Blot Analysis

Total cell lysates were prepared for Western blot analysis as previously described [17]. Briefly, cells were lysed with RIPA buffer containing proteases and phosphatase inhibitors. Equal amounts of proteins were separated by SDS polyacrylamide gel electrophoresis, blotted to PVDF membranes and then transferred using the Trans-Blot Turbo Transfer System (Bio-Rad) with 25 V, 1.0 A, for 30 min. After incubation with 5% non-fat milk (Biorad), membranes were probed overnight with specific primary antibodies (listed in Table S2). Then, the membranes were exposed to secondary horseradish peroxidase (HRP) conjugated antibodies (Bio-Rad) and proteins were detected by the enhanced chemiluminescence system using a ChemiDoc™ XRS+ imaging system (Bio-Rad).

### 2.9. Immunofluorescence Analysis

For immunofluorescence analysis, cells were plated in 6-well plates containing coverslip. After treatment (if present), cells were processed as follows: fixation in 4% formaldehyde/PBS (15 min at room temperature, RT), permeabilization in 0.5% *v*/*v* Triton X-100/PBS (10 min at RT) and blocking in 5% *v*/*v* goat serum and 0.1% *v*/*v* Triton X-100/PBS (1 h at RT). Immunofluorescence staining was performed with overnight incubation of the following antibodies: anti-STAT1 (1:200, Cell Signaling Technology), anti-STAT3 (1:1000, 124H6, Cell Signaling) and anti-Cleaved Caspase-3 (1:400, 5A1E, Cell Signaling Technology). Then, cells were washed twice in PBS and incubated with secondary antibody anti-mouse Alexa Fluor-488 conjugate or anti-rabbit Alexa Flu-or-488 or Alexa Fluor-567 conjugate (1:200) (Thermo Fisher Scientific) for 1 h at RT in the dark. An antifade mounting reagent containing DAPI was used to mount the coverslip onto the slide. Slides were observed under a fluorescence microscope (Carl Zeiss microscopy Gmbh, Jena, Germany) using a 20× or 40× objective.

### 2.10. Kaplan–Meier Plotter Database Analysis

The Kaplan–Meier plotter is an online database integrating gene expression data and clinical information (https://kmplot.com accessed on 12 April 2023) [25]. To evaluate the prognostic value of STAT1 mRNA expression in ovarian cancer, STAT1 (Affymetrix ID 200887_s_at) was entered into this database to obtain the Kaplan–Meier survival plots. Patients with grades 2 and 3 serous carcinoma, stages 3 and 4, were selected for analysis (n = 942 for PFS and 975 for OS). Moreover, analysis was restricted according to the optimal (n = 472 for PFS and 495 for OS) or suboptimal (n = 316 for PFS and 326 for OS) debulking status. The patient’s samples were split into two groups using the “auto-select best cutoff” tool. The hazard ratio with 95% confidence interval and log-rank *p* value were calculated, and survival curves were displayed on the webpage. The ROC plotter (http://www.rocplot.org/ accessed on 27 September 2022) [26] was used to link STAT1 expression (Affymetrix ID 200887_s_at) and response to therapy (relapse-free survival at 12 months) in a population of grade 3, stage 3 serous ovarian cancer patients, treated with Platin and Taxane.

### 2.11. Statistical Analysis

Data presented are representative of three or more independent experiments, unless otherwise stated in the figure legend, and are expressed as mean ± SD. Unpaired Student’s *t*-test (or unpaired *t*-test with Welch’s correction for unequal variances) was used to analyze and compare the means. A statistically significant difference was considered when *p* ≤ 0.05. Biostatistical analysis was carried out using GraphPad software, version 9.3.1 (GraphPad Prism, La Jolla, CA, USA).

## 3. Results

### 3.1. Characterization of Targets of Interest in Different HGSOC Cells

**Figure 1:**
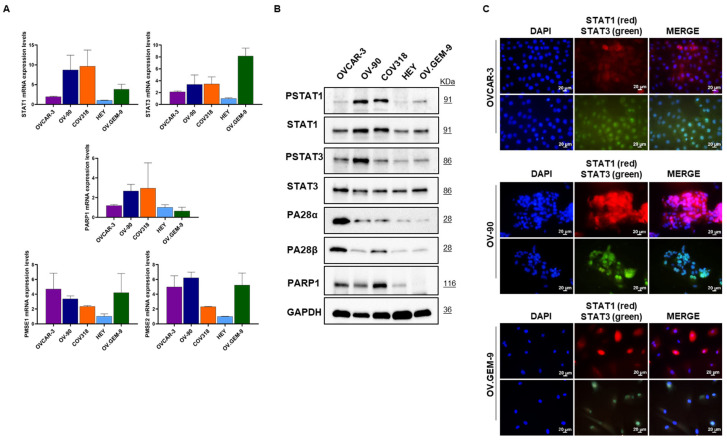
Characterization of targets of interest in high-grade serous ovarian cancer (HGSOC) cells. (**A**) Relative mRNA expression level of STAT1, STAT3, PMSE1 (i.e., PA28α protein), PMSE2 (i.e., PA28β protein) and PARP1 were evaluated by RT-qPCR in a panel of HGSOC cells (OVCAR-3, OV-90, COV318, HEY and OV.GEM-9 cells). Results are presented as fold change compared to HEY cells, as reference sample control. (**B**) Representative Western blot analysis of phospho-STAT1 (Tyr701), STAT1, phospho-STAT3 (Tyr705), STAT3, PA28α, PA28β, PARP1 proteins in OVCAR-3, OV-90, COV318, HEY and OV.GEM-9 cells. Whole cell lysates (20 μg) were loaded into SDS-PAGE, followed by Western blot with specific antibodies. GAPDH was used as control. Blots are representative of two independent experiments. (**C**) Representative pictures showing immunolocalization of STAT1 and STAT3 in OVCAR-3, OV-90 and OV.GEM-9 cells (magnification 40×, bar = 20 µm).

A panel of HGSOC cells, including four established (i.e., OVCAR-3, OV-90, COV318 and HEY) and a primary (i.e., OV.GEM-9) cell line, was characterized in terms of the expression of mRNA and proteins of our interest, i.e., STAT1, STAT3, PA28α/β, and PARP1 (Figure 1A,B). Results obtained showed that OV-90 and COV318 cells expressed the highest STAT1 levels (and protein activation) compared to the remaining cell lines (Figure 1A,B). OV-90 cells also showed the highest STAT3 activation among the tested cells. PARP1 levels were lower in HEY and OV.GEM-9 with respect to the other cell lines. Finally, low protein levels of both PA28 subunits, were observed in all but OVCAR-3 cells. Immunofluorescence of selected representative cells showed the different STAT1/STAT3 protein expression (Figure 1C).

### 3.2. STAT1-Mediated Modulation of PARP1 Levels

**Figure 2:**
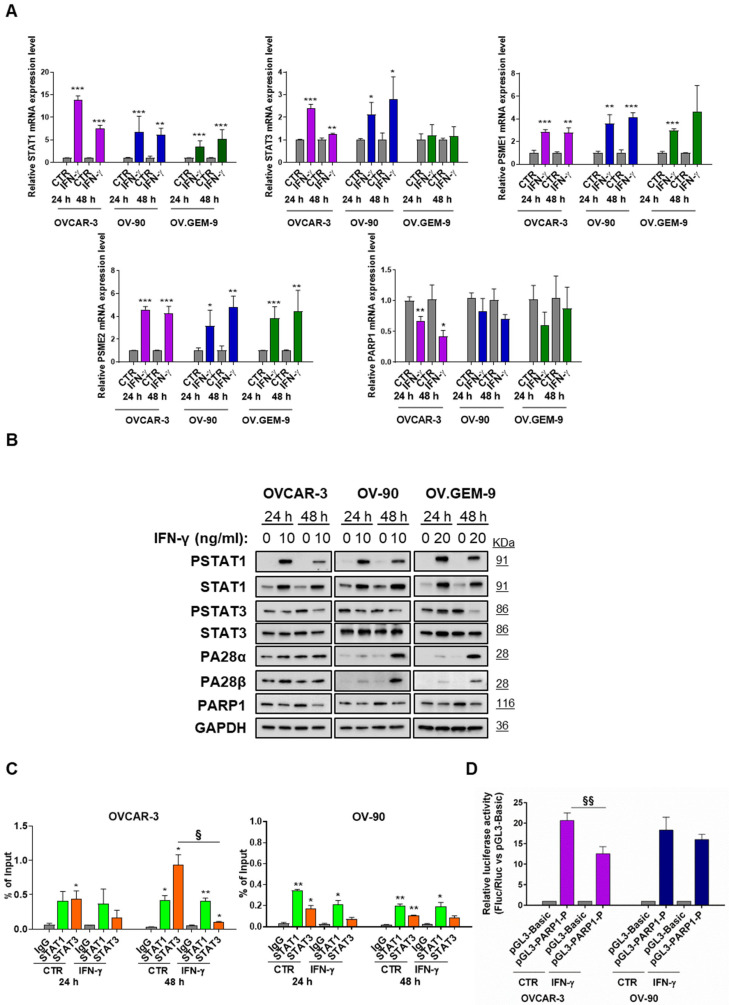
Modulation of PARP1 levels through STAT1. (**A**) Relative mRNA expression levels of STAT1, STAT3, PMSE1 (i.e., PA28α protein), PMSE2 (i.e., PA28β protein) and PARP1 were measured by RT-qPCR after 24 and 48 h of IFN-γ treatment (10 ng/mL for OVCAR-3 and OV-90, 20 ng/mL for OV.GEM-9) (mean ± SD, n = 3). GAPDH was used as reference gene and untreated cells (CTR) were taken as reference sample for each time point. Statistical differences were analyzed by unpaired *t*-test. * *p* < 0.05, ** *p* < 0.01 and *** *p* < 0.001 (IFN-γ vs. CTR). (**B**) Expression levels of phospho-STAT1 (Tyr701), STAT1, phospho-STAT3 (Tyr705), STAT3, PA28α, PA28β, and PARP1 proteins were evaluated by western blot analysis in OVCAR-3, OV-90 and OV.GEM-9 cells. GAPDH was taken as loading control. A representative panel of three independent experiments is shown. (**C**) Specific recruitment of STAT1 and STAT3 at the PARP1 promoter is expressed as percentage of input showing the amount of precipitated DNA (mean ± SD, n = 2). ChIP-qPCR was performed after 24 h and 48 h of IFN-γ treatment (10 ng/mL) in OVCAR-3 and OV-90. Statistical differences were analyzed by unpaired *t*-test. * *p* < 0.05, and ** *p* < 0.01 (IFN-γ group vs. CTR group). § *p* < 0.05 (IFN-γ vs. CTR). (**D**) Relative luciferase activity of PARP1 promoter in OVCAR-3 and OV-90 cells. Cells were transfected with pGL3-Basic or pGL3-PARP1-P plus pRL-TK vector (Renilla luciferase control reporter vector) as an internal control. After 24 h, transfected cells were treated with 10 ng/mL IFN-γ and, after additional 24 h, analyzed. Promoter activity is expressed as ratio of Firefly to Renilla luciferase activity (Fluc/Rluc) normalized to the pGL3-Basic vector (mean ± SD, n ≥ 3). Statistical differences were analyzed by unpaired *t*-test. §§ *p* < 0.01 (IFN-γ vs. CTR).

OVCAR-3, OV-90 and OV.GEM-9 were then chosen to evaluate the effects of STAT1 modulation on STAT3, PA28α/β and PARP1 expression. To this end, cells were treated with IFN-γ in order to induce the activation of STAT1 (10 ng/mL for OVCAR-3 and OV-90, 20 ng/mL for OV.GEM-9). As shown in Figure 2A,B, this treatment caused an induction of STAT1 gene expression at both the mRNA and protein levels, along with its phosphorylation in the amino acid residue of Tyr701. Conversely, STAT3 underwent a dephosphorylation in Tyr705 (an important residue for the transcriptional activity of this transcription factor), not accompanied by a substantial change in the total protein levels. IFN-γ also significantly upregulated the two inducible subunits of the proteasome, PA28α and PA28β, in all the cell lines we used, although at a greater extent in OV-90 and OV.GEM-9 cells. A concomitant decrease in PARP1, at both mRNA and protein levels, was evident in both OVCAR-3 and OV.GEM-9 with a different degree after 24 h and/or 48 h (Figure 2A,B and Figure S1). On the other hand, OV-90 only showed a slight reduction in PARP1 at the protein level as a possible result of the significant increase in STAT1-mediated proteasome induction (Figure 2B and Figure S1). This latter finding is in line with available evidence suggesting that STAT1 regulates proteasome activity by directly modulating proteasome regulators PA28α and PA28β that, in turn, may control PARP1 levels in unchallenged conditions [17].

To investigate the mechanisms underpinning these IFN-γ-related changes, we performed a ChIP-qPCR analysis of the PARP1 proximal promoter, using antibodies directed to the STAT1 and STAT3 proteins. Indeed, using the MatInspector program (Genomatix software suite v3.12), we previously demonstrated the existence of two binding sites for STAT1/STAT3 in proximity of the transcription start site of the human PARP1 gene [17]. Data obtained showed that in OVCAR-3 cells, both STAT1 and STAT3 were bound to the PARP1 promoter in basal condition (Figure 2C). Treatment with IFN-γ induced a significant decrease in STAT3 binding on the PARP1 proximal promoter after 48 h, without change in STAT1 binding (Figure 2C). On the other hand, in OV-90, treatment with IFN-γ did not significantly change the binding of both STAT1 and STAT3 to the promoter region of PARP1 (Figure 2C). These results were corroborated by the luciferase reporter assay. In OVCAR-3, the PARP1 promoter construct showed increased activity compared with the negative control vector, and this activity was reduced by IFN-γ (Figure 2D). Conversely, in OV-90, the activity of the construct was not modulated by IFN-γ treatment (Figure 2D).

**Figure 3:**
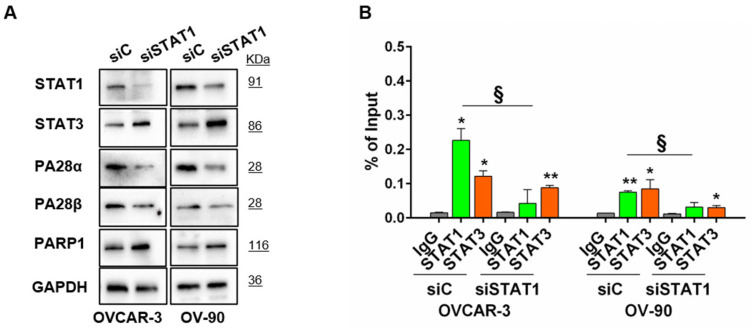
Effects of STAT1 silencing. (**A**) Expression levels of STAT1, STAT3, PA28α, PA28β, and PARP1 proteins were evaluated by Western blot analysis in STAT1-silenced OVCAR-3 and OV-90 cells. After 48 h of silencing, cells were harvested and lysed for protein extraction. Equal amounts of proteins (20 μg) were subjected to Western blot analysis using specific antibodies. GAPDH was taken as loading control. siC: non targeting control siRNA; siSTAT1: STAT1 targeting siRNA. A representative panel of three independent experiments is shown. (**B**) Specific recruitment of STAT1 and STAT3 at the PARP1 promoter is expressed as percentage of input showing the amount of precipitated DNA (mean ± SD, n = 2). ChIP-qPCR was performed after 48 h of STAT1 silencing. Statistical differences were analyzed by unpaired *t*-test. * *p* < 0.05 and ** *p* < 0.01, (siC or siSTAT1 group vs. IgG group). § *p* < 0.05 (siSTAT1 vs. siC).

To further confirm these outcomes, we transiently silenced STAT1 gene in both cell lines. STAT1 silencing produced a slight increase in STAT3 and PARP1 protein levels and a slight decrease in PA28α and/or PA28β proteins in both cell lines (Figure 3A and Figure S2). As expected, ChIP-qPCR analysis after 48 h silencing showed a significant decrease in STAT1 binding on the PARP1 proximal promoter (Figure 3B).

### 3.3. STAT1 Activation Sensitized HGSOC Cells to Cytotoxic Agent

**Figure 4:**
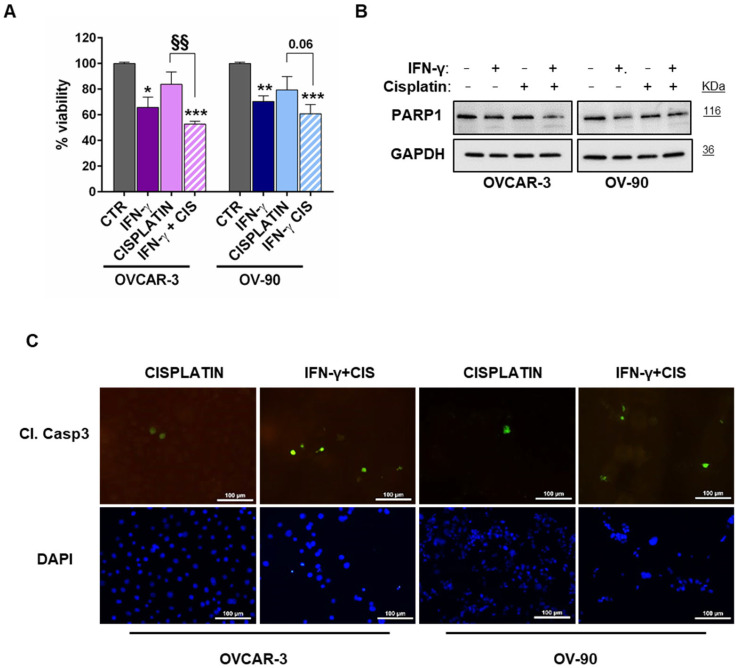
STAT1 activation sensitized high-grade serous ovarian cancer (HGSOC) cells to cytotoxic agent. (**A**) Bar chart representing proliferation assay of OVCAR-3 and OV-90 cells treated with IFN-γ (10 ng/mL) or cisplatin (IC50, 3 µM) as single treatments or in combination compared to respective control cells. For the combination, cells were pre-treated for 24 h with IFN-γ, and then cisplatin was added for a further 24 h (mean ± SD, n = 3). Statistical significances were evaluated through an unpaired *t*-test. * *p* < 0.05, ** *p* < 0.01 and *** *p* < 0.001 respect to CTR. §§ *p* < 0.01 combined treatment with respect to cisplatin. (**B**) Representative Western blot showing modulation of PARP1 levels by IFN-γ with or without cisplatin. Cells were treated as specified above. At the end of treatment, cells were harvested, and whole cell lysates (20 μg) were loaded into SDS-PAGE, followed by Western blot with specific antibodies. GAPDH was used as control. Blots are representative of three independent experiments. (**C**) Representative pictures showing expression of Cleaved Caspase-3 (Cl. Casp3) following treatment with cisplatin alone or in combination with IFN-γ. Cells were treated as specified above, processed for immunofluorescence staining and observed under a fluorescence microscope (magnification 20×, bar = 100 μm).

To begin evaluating the potential clinical relevance of our findings, we tested whether by decreasing PARP1 levels, IFN-γ combination with cisplatin could significantly enhance cytotoxicity in HGSOC cells (Figure 4A). To this end, cells were pre-treated for 24 h with IFN-γ (10 ng/mL) and then treated with cisplatin for further 24 h (3 µM, ≈IC50 at 72 h for both OVCAR-3 and OV-90 cells) [23]. As expected, the effect of cisplatin alone was lower at 24 h of treatment than after 72 h, when the drug’s concentration used reaches its IC50. Importantly, we found that, with respect to cisplatin alone, the combined treatment cisplatin/IFN-γ was able to further decrease cell survival from 83.8 ± 9.6% to 52.8 ± 2.2% (mean ± SD) in OVCAR-3 and from 79.5 ± 10.4% to 60.8 ± 7.2% (mean ± SD) in OV-90 cells, respectively. These changes were accompanied by a reduction in PARP1 levels, observed, in both cell lines, after IFN-γ treatment alone or in combination with cisplatin (Figure 4B). In line with this result, an increase in cleaved caspase-3 protein levels was evident after the treatments, with a greater extent in the combined cisplatin/IFN-γ group (Figure 4C).

### 3.4. STAT1 as a Prognostic/Predictive Factor in HGSOC

**Figure 5:**
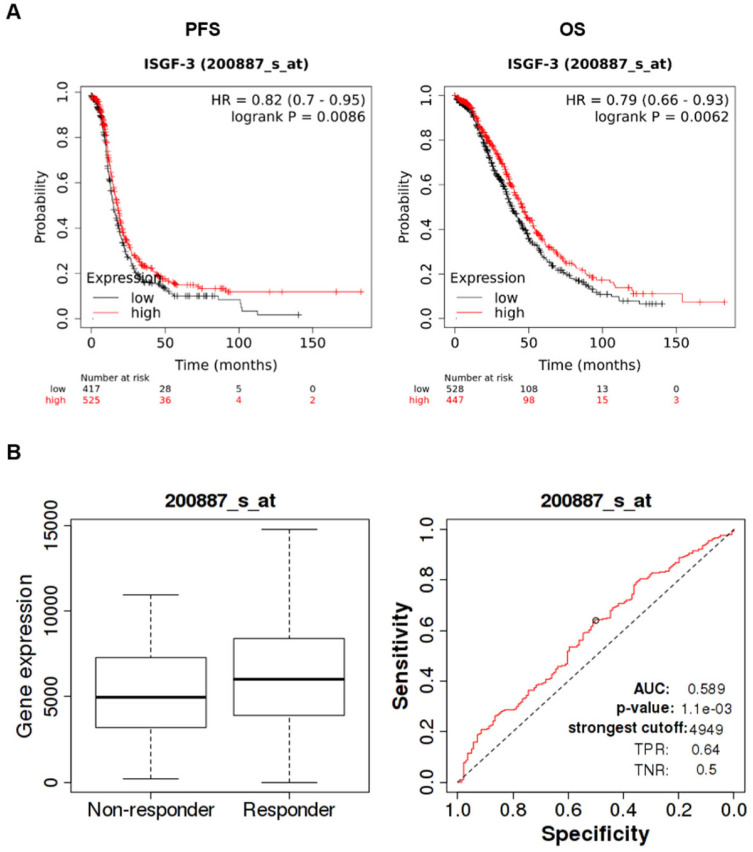
STAT1 (alias ISGF-3) expression and clinical outcome in high-grade serous ovarian cancer (HGSOC) patients. (**A**) The Kaplan–Meier Plotter was used to evaluate the prognostic value of STAT1 (i.e., ISGF-3) (Affymetrix ID 200887_s_at) expression in serous ovarian cancer patients, selected for grade 2 and 3 carcinoma, stage 3 and 4; n = 942 for PFS (progression-free survival) and n = 975 for OS (Overall Survival). *p*-value in the plot represents the result of log-rank test. (**B**) The ROC plotter was used to link STAT1 expression (Affymetrix ID 200887_s_at) and response to therapy (relapse-free survival at 12 months) in a population of grade 3, stage 3 serous ovarian cancer patients, treated with platinum and taxane. Statistical significance of differences in gene expression levels in responders (n = 297) and non-responders (n = 141) was evaluated by the Mann–Whitney test (on the left, *p* = 0.0028). ROC curve analysis shows the sensitivity and specificity of STAT1 in predicting the patient response to treatment.

As all our data indicated that STAT1 might play an essential role in HGSOC progression by controlling PARP1, the major target of PARPi, the KM plotter tool was used to explore its prognostic/predictive role in HGSOC patients. Results obtained are shown in Figure 5A and demonstrated that high STAT1 (alias ISGF-3) expression is associated with better PFS (n = 942, HR = 0.72, 95% CI 0.7–0.95, *p* = 0.009) and OS (n = 942, HR = 0.79, 95% CI 0.66–0.93, *p* = 0.0062) in patients with advanced-stage HGSOC. Notably, ROC Plotter analysis revealed high STAT1 expression to be associated with response to platinum–taxane therapy in the same clinical setting (141 non-responders, 297 responders), AUC 0.589, *p* = 0.001 (Figure 4B). We also performed Kaplan–Meier survival analysis filtering for the debulking status since suboptimal debulking surgery correlates with poor survival in advanced stage ovarian cancer [27]. Results obtained showed that high STAT1 expression is associated with better PFS and OS only in suboptimally debulked patients (Figure S3A), a finding not evident in optimally debulked patients (Figure S3B).

## 4. Discussion

Here, we demonstrated a critical role for IFN-γ/STAT1/STAT3 signaling in regulating PARP1 levels in HGSOC cells, in line with our recent results in cervical cancer [17], and with the emerging evidence on the functional interplay between the STATs signaling cascade and PARP1 [28,29,30,31].

Specifically, results from the present study support a model in which STAT1 and STAT3 control PARP1 gene transcription in HGSOC cells. We also show a role of STAT1-mediated proteasome induction in lowering PARP1 levels. Interestingly, the balance between these two levels of control appears to be the result of the basal expression levels/activation of STAT1/STAT3, as evidenced by the different responses to IFN-γ in the experimental models used. Indeed, the results obtained indicate that while HGSOC cells with low levels of STAT1 respond to the stimulus by activating both transcriptional and post-transcriptional pathways of regulation, only a post-transcriptional control is operating in the presence of high basal STAT1 expression levels. Notably, a similar outcome was observed in cervical cancer cells in our previous study [17], this suggesting a common way of regulation independent of tumor origin.

Of note, it has become increasingly clear that PARP1 may act as a crucial regulator of STAT1/STAT3 transcriptional activity, although the results are not consistent. Specifically, it has been reported that when PARP1 is aberrantly activated in cancer cells, it blocks phosphorylation of STAT1 and STAT3 via the poly(ADP-ribosyl)ation, ultimately reducing their transcriptional activity [28,31]. In line with these findings, Martincuks and colleagues [30] showed that PARPi treatment promotes STAT3 activation in ovarian cancer cells, tumor-associated immune cells and fibroblasts, resulting in PARPi resistance and immunosuppression. On the other hand, a recent paper by Gupte and colleagues [29] suggested that, in macrophages, PARP1-mediated ADPRylation of STAT1α is a driver of the IFNγ-dependent transcriptional program. Overall, these available literature data point to a complex picture of regulation possibly shaped by the cellular context.

The translational relevance of our findings is corroborated by the significant decrease in HGSOC cell viability observed when combining IFN-ɣ with cisplatin, compared to cisplatin alone, an effect accompanied by a marked reduction in PARP1 protein levels. Notably, a recent paper presented evidence that PARP1 overexpression is a near-ubiquitous feature of both primary and recurrent BRCA1/2 mutation-associated tumors, independent of the amplification status [32]. These findings highlight the importance of investigating pathways of PARP1 synthesis and degradation in order to gain insight into the mechanism driving PARPi resistance, finally improving personalized therapeutic approaches in HGSOC.

In line with our hypotheses, Kaplan–Meier/ROC plotter database interrogation suggested that the high STAT1 expression is associated with a better prognosis and response to platinum–taxane therapy in a selected series of HGSOC patients. These findings are in keeping with previous literature data showing a favorable prognostic role of STAT1 in this clinical setting [14,33,34]. Interestingly, our analysis also suggest that suboptimally debulked advanced-stage HGSOC patients could represent the population that mostly benefits from biomarker evaluation.

## 5. Conclusions

Increasing knowledge of the molecular complexity of HGSOC biology may implement the definition of new therapeutic targets, with potentially relevant clinical implications. Although there is much to be further explored, here we demonstrate for the first time that STAT1 might act at both transcriptional and post-transcriptional levels to modulate the PARP1 level in HGSOC, suggesting new pathways to be exploited for targeting cancer cells, based on their dependency on PARP1.

## Data Availability

All data generated or analyzed during this study are included in this published article (and its Supplementary Information Files).

## Supplementary Materials

The following supporting information can be downloaded at: https://www.mdpi.com/article/10.3390/cancers15082361/s1, Table S1: Primer sequences used for RT-qPCR; Table S2: List of Antibodies used in Western Blot analysis; Figure S1: Relative Western Blot quantification of PARP1 to GAPDH in OVCAR-3, OV-90 and OV.GEM-9, at the indicated time points and IFN-γ concentrations, as shown in Figure 2B; Figure S2: Relative Western Blot quantification of PARP1 to GAPDH in OVCAR-3 and OV-90, after 48h of STAT1 silencing, as shown in Figure 3A; Figure S3: STAT1 (alias ISGF-3) determines different clinical outcome in suboptimally or optimally debulked high-grade serous ovarian cancer (HGSOC) patients.
