# Supplementary material for: Exploring the Control of PARP1 Levels in High-Grade Serous Ovarian Cancer
Source: Cancers (Basel). 2023 Apr 18;15(8):2361. doi: 10.3390/cancers15082361 (PMC10137242; doi:10.3390/cancers15082361)

Supplementary Material

Exploring the Control of PARP1 Levels in High-Grade Serous Ovarian Cancer

Giuseppina Raspaglio [1,2], Marianna Buttarelli [1,2,*], Natalia Cappoli [1,2], Alessandra Ciucci [1,2], Anna Fagotti [2,3], Giovanni Scambia [2,3] and Daniela Gallo [1,2,*]

[1] Unità di Medicina Traslazionale per la Salute della Donna e del Bambino, Dipartimento Scienze della Salute della Donna, del Bambino e di Sanità Pubblica, Fondazione Policlinico Universitario A. Gemelli, IRCCS, 00168 Roma, Italy

[2] Dipartimento Universitario Scienze della Vita e Sanità Pubblica—Sezione di Ginecologia ed Ostetricia—Università Cattolica del Sacro Cuore, 00168 Roma, Italy

[3] Dipartimento Scienze della Salute della Donna, del Bambino e di Sanità Pubblica, Fondazione Policlinico Universitario A. Gemelli, IRCCS, 00168 Roma, Italy

* Correspondence: marianna.buttarelli1@unicatt.it (M.B.); daniela.gallo@unicatt.it (D.G.)

This Supplemental material contains:

Supplemental Table S1-S2 and Supplemental Figure S1--S3

Supplementary Table S1. Primer sequences used for RT-qPCR.

Gene Symbol	Primer Forward	Primer Reverse	Amplicon Length (bp)
STAT1	ATGCTGGCACCAGAACGAA	GCTGGCACAATTGGGTTTCAA	84
STAT3	GAGAAGGACATCAGCGGTAA	CAGTGGAGACACCAGGATAT	177
PA28α	CCACACCAAGCTAGAAGGCT	AGCATTGCGGATCTCCATGAC	138
PA28β	CTTTTCCAGGAGGCTGAGGAAT	ATCATCCTTGGGTGGAGGGT	158
PARP1	GCCCTAAAGGCTCAGAACGA	CTACTCGGTCCAAGATCGCC	141
GAPDH	CCTGACCTGCCGTCTAGAAA	CTCAGTGTAGCCCAGGATGC	103

Supplementary Table S2. List of Antibodies used in Western Blot analysis.

Antibody name	Clone	Supplier
Phospho STAT1 (Tyr701)	Polyclonal	Santa Cruz Biotechnology (Santa Cruz, CA)
STAT1	Polyclonal	Cell Signaling Technology (Boston, MA)
PA28α	AT12H3	Santa Cruz Biotechnology
PA28β	G-10	Santa Cruz Biotechnology
PARP1	46D11	Cell Signaling Technology
Phospho STAT3 (Tyr705)	D3A7	Cell Signaling Technology
STAT3	124H6	Cell Signaling Technology
GAPDH	6C5	Abcam

Supplementary Figure S1. Relative Western Blot quantification of PARP1 to GAPDH in OVCAR-3, OV-90 and OV.GEM-9, at the indicated time points and IFN-γ concentrations, as shown in figure 2B. Plots refer to three different experiments of which the more representative is shown in figure 2B.

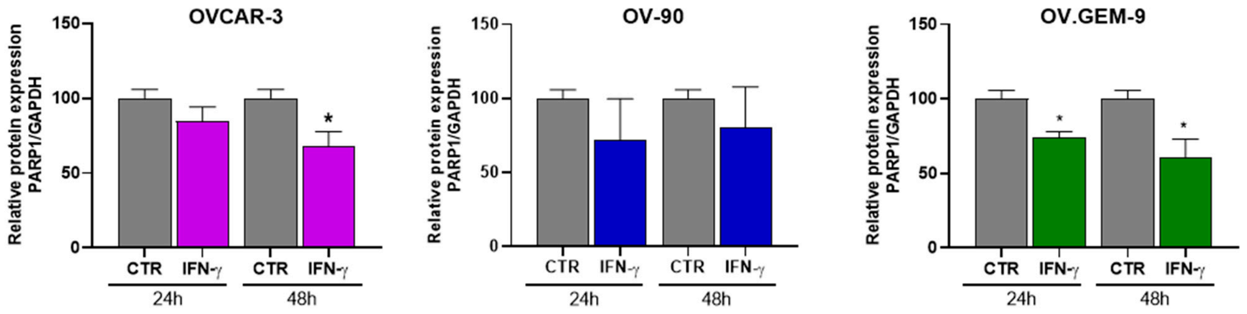

Supplementary Figure S2. Relative Western Blot quantification of PARP1 to GAPDH in OVCAR-3 and OV-90, after 48 h of STAT1 silencing, as shown in figure 3A. Plots refer to three different experiments of which the more representative is shown in figure 3A.

A **OVCAR-3**

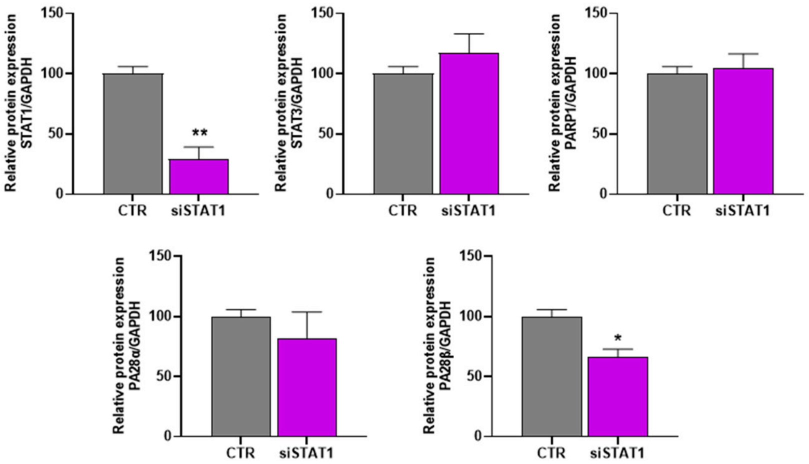

B **OV-90**

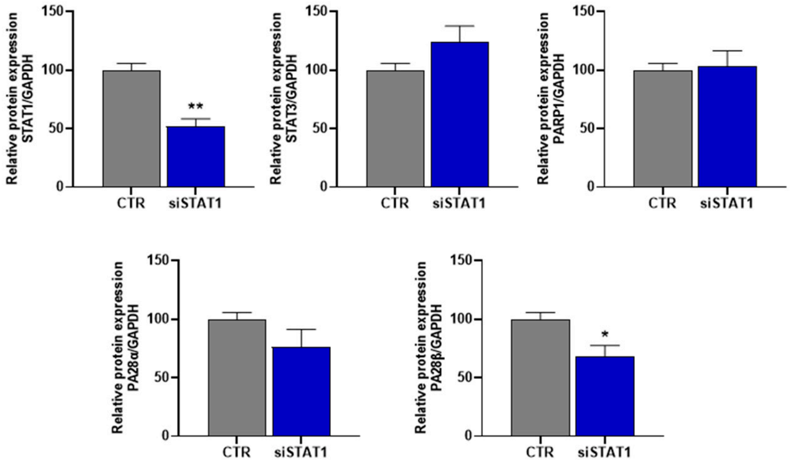

Supplementary Figure S 3. STAT1 (alias ISGF-3) determines different clinical outcome in suboptimally or optimally debulked high-grade serous ovarian cancer (HGSOC) patients. The Kaplan–Meier Plotter was used to evaluate the prognostic value of STAT1 (i.e. ISGF-3) (Affymetrix ID 200887_s_at) expression in serous ovarian cancer patients, selected for grade 2 and 3 carcinoma, stage 3 and 4; debulk: (A) suboptimal; n=316 for PFS (Progression-free survival) and n=326 for OS (Overall Survival); (B) optimal; n=472 for PFS and n=495 for OS. P-value in the plot represents the result of log-rank test.

A) **PFS** **OS**

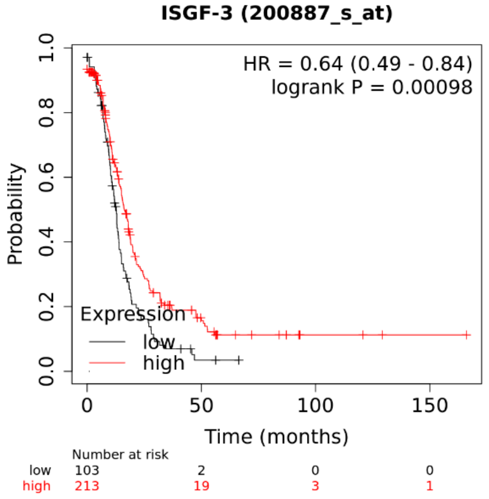

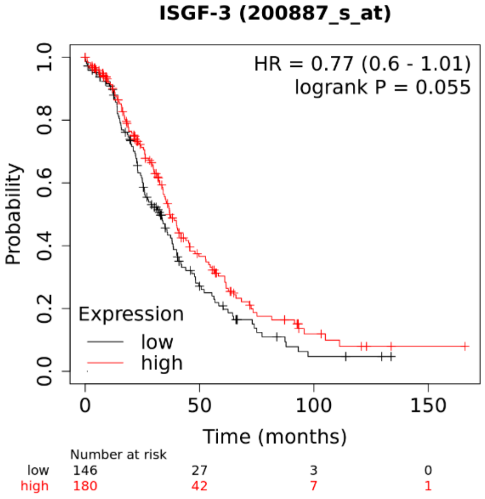

B) **PFS** **OS**

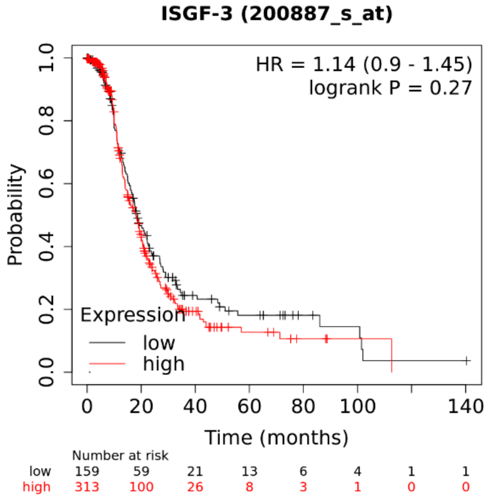

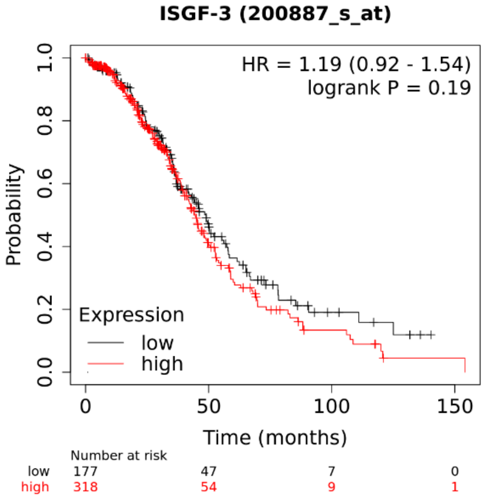

Supplement: Supplementary file 1 [file cancers-15-02361-s001.zip › cancers-2280341-SI.pdf]
